# Tranexamic acid use during total hip arthroplasty: A single center retrospective analysis

**DOI:** 10.1097/MD.0000000000010720

**Published:** 2018-05-25

**Authors:** Nicoleta Stoicea, Kenneth Moran, Abdel-Rasoul Mahmoud, Andrew Glassman, Thomas Ellis, John Ryan, Jeffrey Granger, Nicholas Joseph, Nathan Salon, Wiebke Ackermann, Barbara Rogers, Weston Niermeyer, Sergio D. Bergese

**Affiliations:** a Department of Anesthesiology; b Department of Orthopaedics, The Ohio State University Wexner Medical Center, Columbus; c Orthopedic One, Dublin; d Center for Biostatistics, Department of Biomedical Informatics, The Ohio State University, Columbus, OH; e Weinberg College of Arts and Sciences, Northwestern University, Evanston, IL; f College of Medicine, The Ohio State University; g Department of Neurological Surgery, The Ohio State University Wexner Medical Center, Columbus, OH.

**Keywords:** antifibrinolytics, blood saving measures (BSM), hematocrit (Hct), hemoglobin (Hb), total hip arthroplasty (THA), tranexamic acid (TXA)

## Abstract

**Background:**

Tranexamic acid (TXA) is an antifibrinolytic agent that has shown promise in reducing blood loss during total hip arthroplasty (THA). Several studies have reported side effects of high-dose TXA administration, including myocardial infarction (MI), thromboembolic events, and seizures. These possible side effects have prevented the widespread adoption of TXA in the surgical community.

**Methods:**

We conducted a retrospective chart review of 564 primary and revision THAs performed at a single academic center. Surgical patients received either no TXA or 1 g IV TXA at the beginning of surgery followed by a second bolus just before the surgical wound closure, at the surgeon's discretion. We analyzed differences in hemoglobin (Hb), hematocrit (Hct), estimated blood loss (EBL), and adverse events in patients receiving TXA versus patients not receiving TXA up to 2 days following surgery.

**Results:**

Significantly higher Hb and Hct values were found across all time points among patients undergoing primary posterior or revision THA who had received TXA. In addition, transfusion rates were significantly decreased in both primary posterior THAs and revision THAs when TXA was administered. Patients who received TXA experienced significantly fewer adverse events than those who did not for all surgery types.

**Conclusion:**

Administration of low-dose intravenous (IV) and intra-articular (IA) TXA does not appear to increase rates of adverse events and may be effective in minimizing blood loss, as reflected by Hb and Hct values following THA.

## 1 Introduction

Patients undergoing total hip arthroplasty (THA) face the risk of high surgical blood loss volumes, in addition to significant hidden blood loss caused by bleeding into tissue and hemolysis.^[1–11]^ Perioperative anemia (<11.0 g/dL for females and <12.0 g/dL for males) is, in turn, associated with increased morbidity and mortality.^[4–10,12–17]^ Allogenic blood transfusion (ABT) to correct perioperative anemia is required in 10% to >32% of THA procedures and carries with it a risk of infection, incited immune responses, transfusion-related acute lung injury (TRALI), transfusion-associated sepsis (TAS), hemolytic transfusion reactions (HTR), cancer reoccurrence, prolonged hospital length of stay (LOS), and renal damage.^[5–9,12,16–19,20–25]^ Postoperative risk of infection is increased by 0.1% per unit transfused, with additional risk in immunocompromised patients.^[4–6,22,24–30]^ From a financial perspective, ABT can also incur costs of well over $1000 during total joint arthroplasty (TJA) procedures.^[7–8,31–34]^

Research into pharmacological modalities of controlling intra- and post-operative bleeding has intensified in recent years. One such pharmacologic area of inquiry is tranexamic acid (TXA) (Cyklokapron (Pfizer, New York, NY)). Upon activation of the fibrinolytic pathway, plasminogen is converted to fibrinolytic plasmin via tissue plasminogen activator (t-Pa). The intravenous (IV) formulation of TXA is a synthetic lysine derivative acting through competitive inhibition of lysine binding sites on plasminogen, thereby reducing the local degradation of fibrin clots by plasmin.^[34–38]^ Consequently, TXA is a prime candidate for minimizing cases of postoperative anemia and, ultimately, decreasing transfusion rates. However, the optimal TXA dosage, whether TXA dosage should be weight based, and the ideal timing and route of TXA administration are subjects of continuous contention, with conflicting study findings present throughout the literature.^[39]^

The IV route of administration remains the enduring clinical standard, although more recent studies have indicated that topical administration (intra-articular [IA]) may be superior in limiting not only overt blood loss but also bleeding into the surrounding tissue.^[40,41]^ In addition, IA TXA has been reported to reduce both inflammation at the surgical site and the risk of TXA's systemic side effects. IA administration of TXA does not affect artificial joint function or wear.^[42]^ Newer studies have begun to investigate the combined usage of IV and IA TXA, with a wide range of dosing protocols and various findings.^[39,43,44]^ Based upon these and several other trials, low-dose IV and IA TXA appears to be safe for use during orthopedic procedures and may be capable of significantly reducing total blood loss, dips in Hb, and transfusion rates.^[6,7,39,45–50]^

To further evaluate the benefits and possible risks of both IV and IA TXA, we conducted a retrospective chart review to compare transfusion rates, complications, and postoperative outcomes of patients at our academic center receiving bolus IV and IA TXA versus no TXA during THA.

## 2 Methods

Following institutional review board (IRB) approval, primary and revision THA cases performed by 5 surgeons from January 2013 through July 2015 were reviewed retrospectively by accessing electronic medical records (EMR). Each patient's chart was examined to determine whether the patient met the inclusion or exclusion criteria.

The review included patients over 18 years of age undergoing either a primary or revision, unilateral THA procedure with preoperative Hb values ≥11 g/dL and normal international normalized ratios (INR), prothrombin times (PT), and partial thromboplastin time (PTT) values. The following conditions were excluded: allergy to TXA, bilateral THA, hepatic dysfunction, chronic renal failure, symptomatic seizure disorders, cerebral infarction, bleeding disorder, hemodialysis requirement, anticoagulant medication, or long-acting nonsteroidal anti-inflammatory drugs (NSAIDs). Patients with a history of ischemic heart disease or chronic heart failure were also excluded.

Patients’ charts were screened for demographics (age, gender, ethnicity, body mass index—BMI, American Society of Anesthesiologists (ASA) score), whether TXA was received and route of administration, Hb and Hct values at baseline and following surgery (postoperative day 1 and 2), blood transfusions, and units of blood transfused. The occurrence of vaso-occlusive events (VOEs) and other adverse events were also recorded.

### 2.1 Surgical management

All procedures took place at The Ohio State University Wexner Medical Center. For patients receiving TXA, a 1 g bolus was administered intravenously at the beginning of surgery. At the end of surgery another 1 g bolus was given by either IV or IA route, according to the surgeon's preference.

Surgeons used either an anterior or posterior approach for primary THA procedures. Postoperatively, a hemovac was connected to the drain positioned at the site of the surgical wound to assist with blood and fluid removal. Based on progress notes, compressive stockings were prescribed for the duration of their postoperative hospital stay. To prevent thrombosis, prophylactic anticoagulant medications were given to patients after surgery. Chart reviews demonstrated a general trend in the threshold for transfusion based on hemoglobin levels. Generally, for patients without cardiovascular disease, blood transfusions were administered for Hb values below 7 g/dL. Patients with cardiovascular disease and patients who did not tolerate low Hb values were transfused at Hb levels of <8 g/dL and <10 g/dL, respectively.

### 2.2 Study design

A total of 564 THA patients were included in the study. EMRs were reviewed to determine whether they had received TXA during surgery. Overall, 394 (69.86%) patients received TXA while 170 (30.14%) patients did. Within the TXA group, 141 patients (35.79%) underwent primary THA by anterior approach, 213 patients (54.06%) underwent primary THA by posterior approach, and 40 patients (10.15%) underwent revision (anterior/posterior approach). Within the no-TXA group, 31 patients (18.24%) underwent primary THA by anterior approach while 114 patients (67.06%) underwent THA by posterior approach, and 25 (14.71%) underwent revision.

### 2.3 Statistical methods

Continuous demographic and clinical variables were summarized and compared between study groups using Student's *t*-tests or Wilcoxon rank sum tests where relevant. Study group differences for categorical variables were tested using chi-square or Fisher's exact tests. Multivariate linear mixed models with random intercepts were fit to compare each of the continuous hemoglobin and hematocrit outcomes between TXA groups for each surgical approach adjusting for potential confounders and other clinical variables and including appropriate interaction terms. *P*-values <.05 were considered to be statistically significant. All statistical analyses were performed in SAS 9.4 (SAS Institute, Cary, NC).

## 3 Results

### 3.1 Demographics

The demographics of TXA and no-TXA groups are presented in Table 1. Patients receiving TXA were younger (56.17 vs 60.01 years, *P = *.0002) than no-TXA patients. The no-TXA patients had higher ASA scores (*P < *.0001) and were more likely to have a history of coagulation pathology (2.94% vs 0.00%, *P = *.0024), COPD (10.00% vs 4.31%, *P = *.0024) and cancer (15.29% vs 6.35%, *P = *.0007) (Table 1). Patients within the no-TXA group were also more likely to be African American or black (17.06% vs 13.71%, *P = *.0974), though the difference between races was not statistically significant.

**Table 1:**
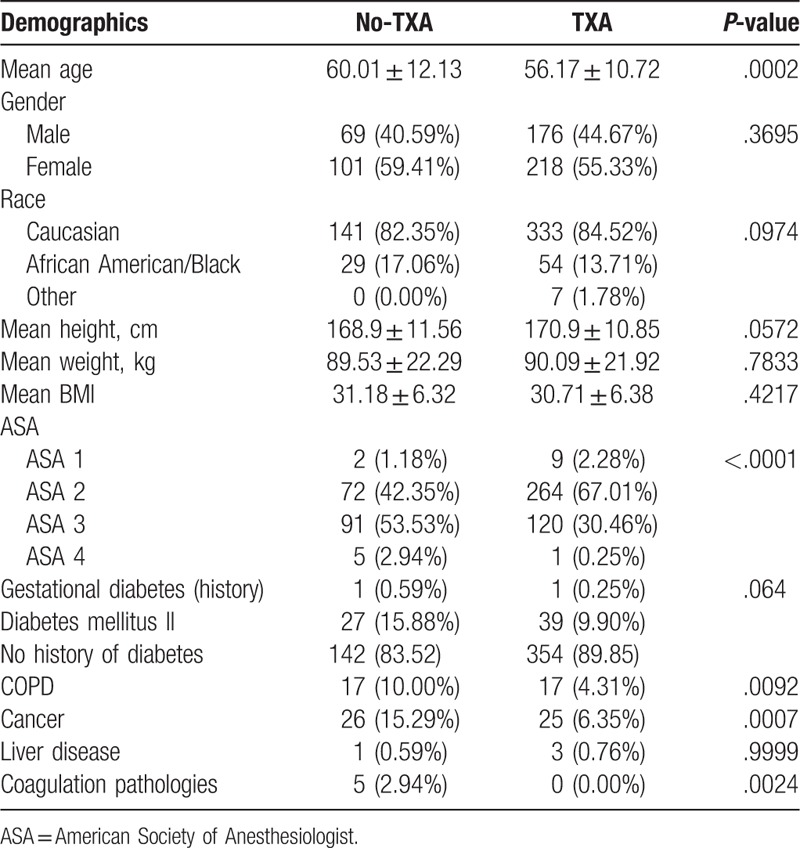
Demographics.

**Figure 1:**
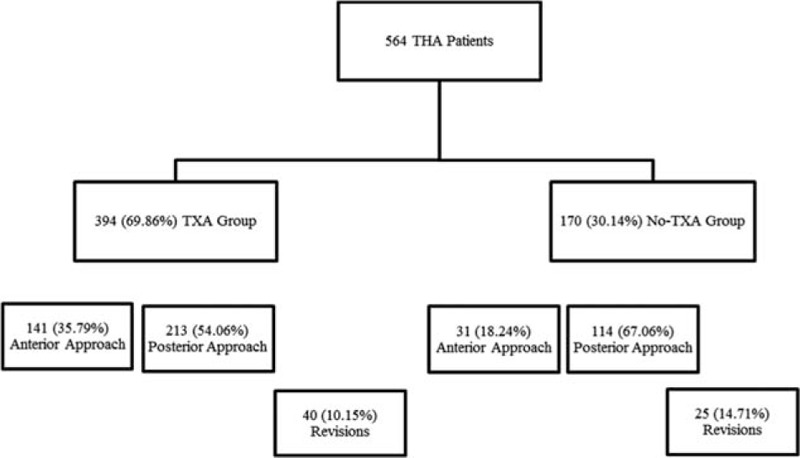
Number of subjects in TXA and no-TXA groups, and surgical approach underwent. TXA = tranexamic acid.

### 3.2 Medications

Concomitant medications taken by patients within the TXA and no-TXA groups are presented in Table 2. TXA group patients were more likely to be maintained on NSAIDs (92.39% vs 51.19%, *P < *.0001) and less likely to be prescribed Lovenox (8.12% vs 28.82%, *P < *.0001) or warfarin (4.06% vs 47.06%, *P < *.0001).

**Table 2:**
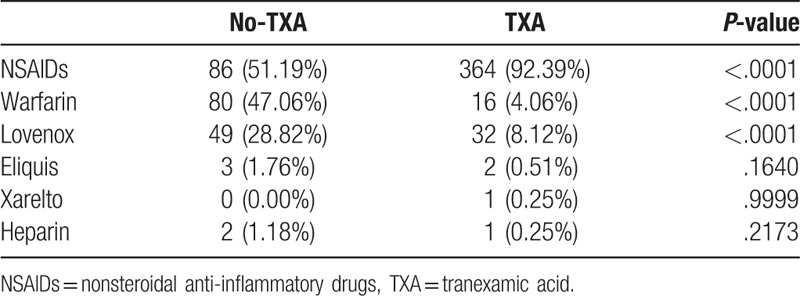
Concomitant medications.

A majority of patients underwent THA under general anesthesia (no-TXA group 96.47% and TXA group 97.46%). A minority received neuraxial block combined with general anesthesia (no-TXA group 2.94% vs TXA group 2.03%). Only 2 patients from the TXA group and 1 patient from the no-TXA group received neuraxial block only (Table 3).

**Table 3:**
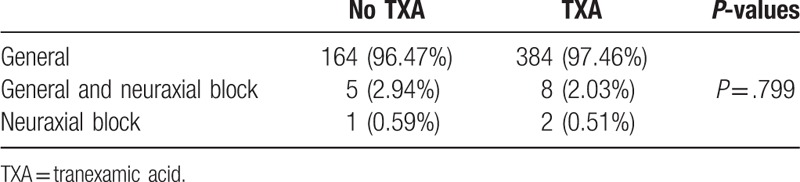
Type of anesthesia.

### 3.3 Blood loss

**Figure 2:**
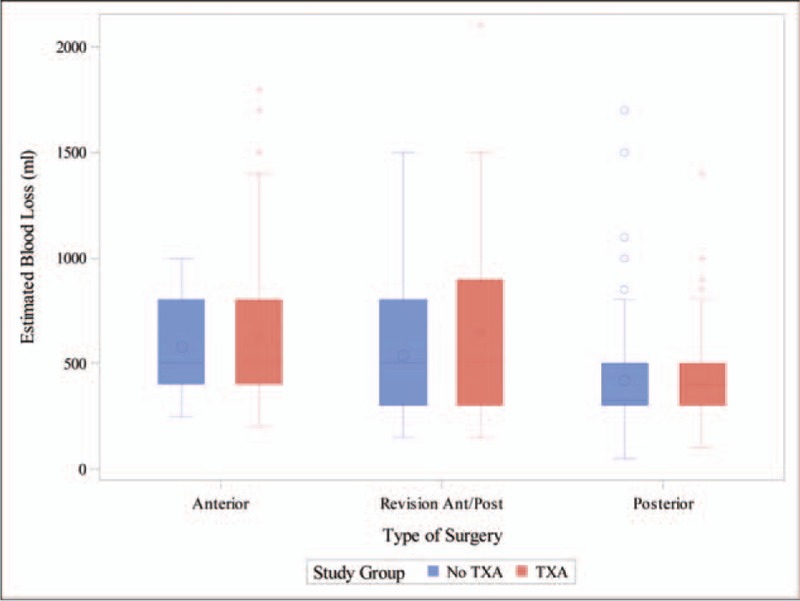
Estimated surgical blood loss between the TXA and no-TXA groups, by surgical approach. TXA Group: 0 = No TXA, 1 = TXA. TXA = tranexamic acid.

With a direct anterior approach, median estimated blood loss (EBL) retrieved from operatory notes was 500.00 (IQR: 400.00, 800.00) mL in both study groups (*P = *.3222); in the postero-lateral approach, median EBL was 400.00 (IQR: 300.00, 500.00) mL for the TXA group and 325.00 (IQR: 300.00, 500.00) mL for the no-TXA group (*P = *.8158); for those undergoing a revision, median EBL was 500.00 (IQR: 300.00, 900.00) mL in the TXA group and also 500.00 (IQR: 300.00, 800.00) mL in the no-TXA group (Fig. 2). Crystalloid volume consumption, operative times, and PO creatinine levels did not differ significantly between TXA and no-TXA groups for any approach (Table 4).

**Table 4:**
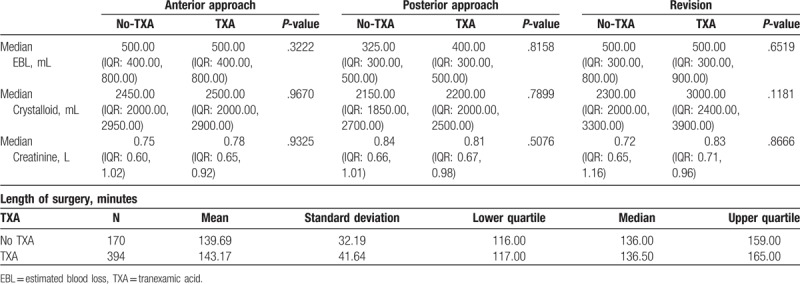
Surgical parameters.

### 3.4 Hemoglobin and hematocrit

**Figure 3:**
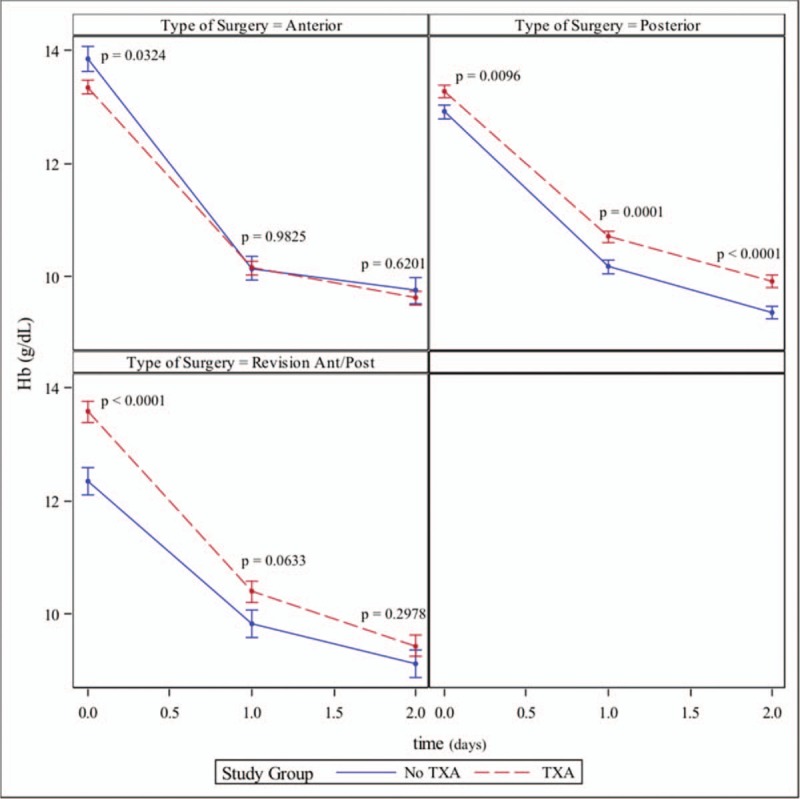
Hemoglobin before and after surgery. Estimates are based on longitudinal multivariate models adjusted for sex, type of surgery, and transfusion (yes/no).

**Figure 4:**
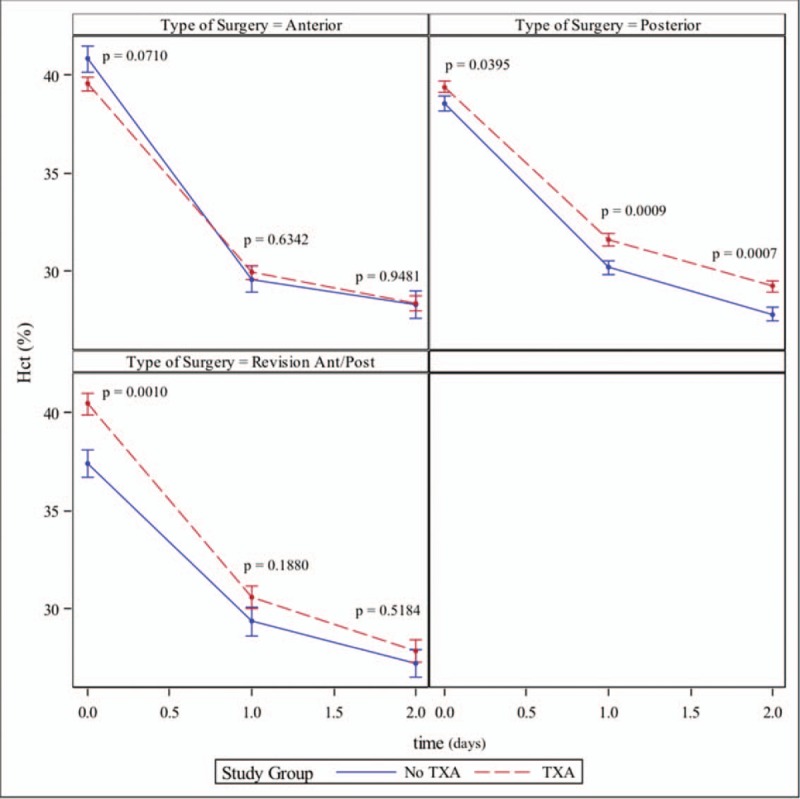
Hematocrit before and after surgery. Estimates are based on longitudinal multivariate models adjusted for sex, type of surgery, and transfusion (yes/no).

Mean hemoglobin and hematocrit measurements following surgery are presented in Figure 3 (hemoglobin) and Figure 4 (hematocrit) for each group, both of which utilize models based estimates of means +/− standard error.

### 3.5 Transfusions

In patients receiving primary THA with a posterior approach, and in those receiving a revision THA, a significant difference in transfusion requirement was observed between the TXA and no-TXA groups (posterior: no-TXA group 22 (19.30%) transfused vs TXA group 6 (2.82%) transfused, *P < *.0001; revision: no-TXA group 15 (60.00%) transfused vs TXA group 11 (27.50%) transfused, *P = *.0093). No significant difference in rate of transfusion was found between TXA groups during THAs with an anterior approach (no-TXA group 5 (16.13%) transfused vs TXA group 12 (8.51%) transfused; *P = *.1981).

For the anterior approach, 6 units were transfused overall in 2 patients within the no-TXA group, and 0 were transfused in the TXA group. Cell saver (270 mL) was used in one patient within the no-TXA group, while Hespan (500 mL) was used in 3 no-TXA patients and 1 TXA patient. Packed red blood cell unit use did not differ significantly between TXA groups for anterior, posterior, or revision THAs.

### 3.6 Adverse events

In the TXA group, 2 (0.51%) primary posterior THA patients experienced MI while 1 primary anterior, 3 primary posterior, and 3 revision patients experienced MI in the no-TXA group (4.12%) (*P = *.0041). More strokes occurred within the no-TXA group than the TXA group (9 (5.29%) vs 3 (0.76%), respectively; *P = *.0016). One seizure occurred, in a patient within the TXA group (0.25%, *P = *.9999) and no seizures occurred in the no-TXA group. One patient in the no-TXA group (0.59%) and 0 patients in the TXA group (0.00%) were diagnosed with a DVT following surgery (*P = *.3014). The rate of PO PE did not differ significantly between groups (TXA group 0 (0.00%) vs no-TXA group 1 (0.59%), *P = *.3014).

Two revision THA patients within the no-TXA group (1.18%) required ICU admission immediately after surgery (*P = *.0904).

## 4 Discussion

Total joint surgeries often lead to high perioperative blood loss, thus requiring ABT and increasing the risk of complication and infection. Restrictive transfusion protocols with hemoglobin (Hb) thresholds as low as 7.0 g/dL have been proposed; however, this strategy is not suitable for all patients.^[9,51–54]^ Therefore, alternative techniques have been developed to reduce ABT, including red blood cell (RBC) salvage, autologous transfusion, local and regional anesthesia, hypotensive epidural anesthesia, controlled hemodilution, and antifibrinolytic administration.^[20,55–58]^

The major findings of our study were that 1 or 2 single gram boluses of TXA (IV or IA) reduced the extent of postoperative Hb and Hct drop following primary posterior and revision THA and did not increase the frequency or severity of adverse events.

IV TXA has a half-life of approximately 2 hours for a 1 g bolus. TXA has a distribution volume of 9 to 12 L and is excreted in the urine with 95% of the dose chemically unaltered. The plasma concentration of TXA required for approximately 80% inhibition of fibrinolysis is 10 μg/mL.^[62]^ For IV dosing, the maximum concentration is achieved about 1 hour after administration and the fibrinolytic effects last from 8 to 17 hours, although pharmacokinetic studies have suggested that age may slightly alter the therapeutic window owing to efficiency of drug clearance.^[63,64]^

A large dosing study by Horrow et al suggested a 10 mg/kg loading dose of TXA followed by a 1 mg/kg/hr infusion. More recent studies, including those by Ker et al and North et al, have explored the use of IA infiltration routes for more targeted administration of TXA.^[45,46]^ Current studies have mainly investigated possible benefits of dual route IV/IA dosing, with positive results in single route administration protocols.^[39,65]^ However, within the orthopedic surgical setting, the standard dosage, administration route, and frequency have yet to be determined.

TXA's efficacy in preventing fibrin-clot degradation, as well as its 90% renal elimination within 24 hours, makes it a prime candidate for use in TJA procedures.^[62]^ In the first hours of surgery, there is an enhanced release of tissue plasminogen factor (t-PA) that remains elevated for up to 24 hours, contributing to increased fibrinolytic activity and, in turn, postoperative anemia. Shortly after this period, the activity of t-PA decreases during a fibrinolytic shutdown phase due to a release of tissue plasminogen activator inhibitor-I (t-PAI), an acute phase reactant that naturally limits fibrinolysis.^[34,37,66,67]^ Thus, the use of TXA can be utilized preoperatively or within the early intraoperative phase to combat the enhanced release of t-PA during surgery. TXA administered more than 24 hours post-surgery may cause over-inhibition of fibrinolytic activity due to the combined effects of TXA and t-PAI, possibly resulting in vaso-occlusive events (VOE) such as deep vein thrombosis (DVT) and pulmonary embolism (PE).^[48,56,68]^

TXA has been demonstrated to improve clotting and reduce blood loss in previously published literature.^[48,56,73,78–80]^ However, reports of dose-dependent adverse events have limited the widespread adoption of TXA within the orthopedic surgical theater.^[7,68–73,81]^ In the present study, we used a low dose (1 g IV) of TXA before the initial incision and a second bolus (1 g IV or IA) before the end of surgery, in accordance with our center's standard of care and with previously published evidence.^[68,82,83]^ Theoretically, this regimen allows for a strong antifibrinolytic effect soon after the beginning of surgery, while the second IA bolus allowing for local microvascular hemostasis prior to closure. At our center, most patients received IV/IV TXA rather than IV/IA.

Our study population consisted of adult patients largely with an American Society of Anesthesiologists (ASA) physical status classification of II or III. Due to variations in blood loss associated with the 3 different types of procedure used (direct anterior, postero-lateral, and revision), analysis of the TXA and no-TXA groups was separated by surgical approach.^[84–89]^ Patients within the TXA treatment cohort tended to be younger and were less likely to have been diagnosed with cancer, COPD, or coagulation pathologies. A larger percentage of the TXA group was maintained on NSAIDs than the no-TXA group, while warfarin and Lovenox (Sanofi S.A., Gentilly, France) were more prevalent in the no-TXA group.

### 4.1 Heparin-vitamin K antagonist bridging for surgery

The 9th practice guidelines from the American College of Chest Physicians (ACCP) provide information on anticoagulation therapy and thromboprophylaxis during major orthopedic procedures including THA and TKA. These guidelines suggest that low-molecular-weight heparin (LMWH) be used 12 hours prior to or 12 hours after major orthopedic procedures in combination with an incremental pneumatic compression device (IPCD) for dual prophylaxis in otherwise healthy patients.^[97]^

In patients maintained on vitamin K antagonists (VTA) such as warfarin, guidelines for anticoagulation therapy and bridging to heparins are separated by thrombotic risk strata (high, medium, and low). The CHADS_2_ scoring method is the most widely used and is one of the few validated scales for both surgical and nonsurgical patient risk. Specifically, patients considered high-risk on the CHADS_2_ scale have a >10%/year chance of a thrombotic event, medium-risk patients have a 5% to 10%/year chance of a thrombotic event, and low-risk patients have a thrombotic risk of <5%/year. At our center, patients maintained on VTA therapy, and those who are considered to be within the “high-risk” stratum, are bridged to heparin preoperatively. Postoperatively, a low-dose of LMWH or aspirin is administered to all patients, regardless of VTA concomitancy or risk of thrombotic events.

Wind et al. (2013/2014) found that TXA improved Hct and Hb levels at discharge in primary THA and TKA.^[90,91]^ While Wind et al concluded that IV TXA produced more predictable and therefore superior results to IA TXA, Wei et al. (2014) found no significant differences between PO THA Hb and Hct improvements over the control group between IV and IA administration routes.^[91,92]^ Yue et al^[93]^ also found significant Hb and Hct drop reductions with IA TXA in primary THA patients. A trend toward increased Hb values on postoperative day 1 was noted in revision patients when compared to patients who did not get TXA; however, this difference failed to reach statistical significance (*P = *.0633). No significant differences between treatment groups were noted in anterior THA patients at any time point. Our study demonstrated significant reductions in decline of Hb and Hct levels only in the posterior approach cohort. Wei et al. included only posterior THA patients while Wind et al's cohort consisted of primarily anterior THA approach patients and Yue et al utilized a posterolateral approach.^[91–93]^

Though our study results did not show evidence of a significant reduction in EBL, crystalloid consumption, or an increase in creatinine within the TXA cohort, this study did demonstrate a significant reduction in transfusion rates for TXA patients undergoing THA with posterior approach or revision when compared with the no-TXA group (*P < *.0001 posterior; *P = *.0093 revision). These findings support those previously published by Konig et al^[94]^ who also reported significant reductions in transfusion rates when TXA was used in primary posterior THA (15% v 1%, *P* < .01), and Shah et al^[95]^ who studied TXA administration in revision THA.

### 4.2 Adverse events

Due to the rarity of these VOE reports in TXA treatment groups, recent publications have reached varying efficacy and safety conclusions when using TXA in THA.^[68]^ Myocardial infarction (MI) and cerebrovascular events have been observed.^[7,68–73]^ Additionally, a small number of studies have noted TXA's potential to induce seizures when accidentally injected intrathecally during spinal anesthesia^[69,74–77]^ or when administered in very large doses during cardiac surgery.^[70–72]^ TXA likely produces these seizures through competitive antagonism of glycine and GABA_A_ receptors and disinhibition within the central nervous system.^[69,74–77,97]^

The current study demonstrated that in a large cohort of orthopedic patients undergoing primary THA or revision with a direct anterior and posterolateral approach, low-dose IV or IA TXA could be given safely without an increase in adverse events. Significantly fewer MIs, strokes, ischemic events, and CHF events occurred within the TXA group versus the no-TXA group. Only 2 patients within the no-TXA group and no patients in the TXA group required immediate PO ICU admission. One of the no-TXA patients had been in the ICU prior to the procedure and returned immediately postoperatively while the second patient suffered respiratory failure postoperatively and required continued mechanical ventilation. In the no-TXA group, 1 DVT and 1 PE occurred. No DVTs or PE events were reported in patients receiving TXA. In all cases, the TXA group experienced equal or lower rates of complications when compared to those patients not receiving TXA.

Only 1 seizure was reported in the TXA cohort, the patient had a history of generalized tonic-clonic seizures and was being maintained on phenytoin (200 mg). This patient had previous laboratory values indicating sub-therapeutic levels of the drug, suggesting poor compliance or ineffective phenytoin treatment. The distribution of these adverse events may be the result of selection bias for which candidates receive TXA. It was noted in the study that patients who did receive TXA were younger and had fewer comorbidities. Because risks of DVT, PE and embolic events are known to be associated with TXA administration, TXA may be withheld from patients with a history of these events or pre-existing coagulopathy. This selection bias places patients with a higher risk of MI, stroke, DVT and PE into the no-TXA group. In addition, the older patients with greater comorbidities are at risk of other adverse events not associated with embolic or coagulopathic pathology.

### 4.3 Limitations

The limitations of this study include its retrospective nature and lack of randomization to account for intergroup differences. This study was also conducted in only adult patients undergoing elective, unilateral THA and the benefits of TXA use may not be translatable to the emergent care setting or to bilateral THA procedures wherein more extensive bleeding is expected. Further studies are needed to examine the risks and benefits of TXA use in pediatric populations. It has also been suggested that age may significantly impact TXA clearance. For this reason, additional pharmacokinetic and dosing studies should be conducted in young as well as elderly populations. Additionally, a cost-benefit analysis was not conducted to determine the cost savings of using TXA during THA to reduce transfusion costs.

The small revision THA cohort size also limited the power of certain findings. Further studies in revision patients are necessary to better determine the benefits of TXA in this patient group.

The fact that this study was conducted at only one center may also limit the generalizability of these results, as transfusion thresholds differ between centers and even between anesthesiologists.

## 5 Conclusion

The data from this cohort suggest that TXA administration reduces transfusion rates following THA, particularly for the posterior approach and revisions. Additional prospective studies should be conducted to determine the optimal dosage, route, and administration time of TXA. Our findings, which are supported by previous orthopedic TXA studies, indicate that low-dose IV and IA TXA should be adopted more extensively in the orthopedic setting as a safe and effective method of reducing perioperative transfusions and their associated risks in otherwise healthy adult patients. An orthopedic meta-analysis and subsequent consensus on the ideal TXA dosing protocol is needed for both IV and, particularly, IA drug routes. TXA clearance and an optimal dosing regimen in the elderly, youth, and patients with impaired glomerular filtration rates (GFR) should also be examined in future studies. In addition, selection bias inherent to a retrospective study may have contributed to the appearance that patients receiving TXA are at a lower risk of MI, stroke, DVT, and ICU admission than those who do not receive TXA. The higher risk patients in the no-TXA group may also correspond with a more complicated surgical course and a higher risk of bleeding, which could also be a future area of study among TXA patients. A prospective study that randomizes patients into TXA and no-TXA groups would prevent bias that results in the withholding of TXA from high-risk patients. However, clotting and embolic complications associated with TXA are well known. Randomizing patients at risk of coagulopathy to receive TXA may not be considered ethical or appropriate. While we found no evidence to indicate an increase in VOEs or other complications, future prospective studies should continue to monitor rates of adverse events previously reported with high-dose TXA.

## Acknowledgments

The authors gratefully acknowledge David Connell, Cassie Lawrence, and Steven Majoribanks for their contributions and collaborations in data collection, result discussion, and editing.

## Author contributions

**Conceptualization:** Nicoleta Stoicea.

**Formal analysis:** Nicoleta Stoicea.

**Investigation:** Nicoleta Stoicea.

**Methodology:** Nicoleta Stoicea.

**Project administration:** Nicoleta Stoicea.

**Supervision:** Nicoleta Stoicea.

**Writing – original draft:** Nicoleta Stoicea.

**Writing – review & editing:** Nicoleta Stoicea.
